# Zn-Alloyed All-Inorganic Halide Perovskite-Based White Light-Emitting Diodes with Superior Color Quality

**DOI:** 10.1038/s41598-019-55228-1

**Published:** 2019-12-09

**Authors:** Saroj Thapa, Gopi Chandra Adhikari, Hongyang Zhu, Alexei Grigoriev, Peifen Zhu

**Affiliations:** Department of Physics and Engineering Physics, The University of Tulsa, Tulsa, OK 74104 United States

**Keywords:** Nanoscience and technology, Optics and photonics

## Abstract

Recently, lead halide perovskite nanocrystals (NCs) have gained tremendous attention in optoelectronic devices due to their excellent optical properties. However, the toxicity of lead limits their practical applications. Here, the synthesis of Zn^2+^-alloyed CsZn_x_Pb_1-x_X_3_ (up to 15%) NCs is reported to achieve lead-reduced white light-emitting diodes (WLEDs). The incorporation of Zn^2+^ into CsPbX_3_ host NCs results in a lattice contraction, without altering the structure and morphology, which has a direct effect on the optical properties. The blue-shifts in the photoluminescence emission and increase in bandgap is observed while retaining high photoluminescence quantum yield. Then by engineering the different compositions of halides for 15% Zn^2+^-alloyed CsZn_x_Pb_1-x_X_3_ NCs, tunable emission (411–636 nm) is obtained. Notably, the WLEDs are experimentally demonstrated employing the lead-reduced NCs (blue, green, yellow, and red). By varying the ratios of the amount of NCs, white lights with a tunable correlated-color temperature (2218–8335 K), an exemplary color-rendering index (up to 93) and high luminous efficacy of radiation (268–318 lm·W^−1^) are obtained. Best of our knowledge, these are superior to other reported WLEDs based on CsPbX_3_ NCs doped with transition metal ions. This work places the halide perovskite NCs one-step closer in designing the environmentally benign and energy-efficient WLEDs.

## Introduction

Semiconducting metal halide-based perovskite nanocrystals (NCs) have been studied extensively over the past several years due to their remarkable optoelectronic properties, low-cost solution processability, and scalable fabrication^1–8^. These superior properties make them promising materials in modern optoelectronic devices such as solar cells, light-emitting diodes (LEDs), photodetectors, and lasers^9–20^. The previous studies reported that all-inorganic lead halide perovskites (CsPbX_3,_ ‘X’-halides) with better moisture and thermal stability in comparison to their organometallic counterparts renders excellent photophysical properties such as high defect tolerance, high photoluminescence quantum yield (PLQY), narrow emission line-widths, and wide color gamut^20,21^. These are desirable for lighting applications, especially for the LEDs. Song *et al*. for the first time, reported the blue, green, and yellow LEDs based on CsPbX_3_ perovskite quantum dots (QDs)^22^. Thereafter, the application of this type of material in white LEDs (WLEDs) is on the verge of rapid development. Pan *et al*. demonstrated a WLED employing the green-emitting CsPbBr_3_-polymethyl methacrylate (PMMA) and red-emitting CdSe/ZnS-PMMA composite film in combination with GaN chips, which yields a luminous efficiency of radiation (LER) of 65 lm·W^−1^ and the CIE color coordinates of (0.31, 0.32)^23^. Bi *et al*. incorporated the red-emitting CsPbX_3_ perovskite QDs and yellow-emitting YAG:Ce phosphor with a commercial blue LED chip to achieve white light emission with a tunable correlated-color temperature (CCT) from 2854 K to 11,068 K, color-rendering index (CRI) of 89, LER of above 84.7 lm·W^−1^, and the CIE color coordinates optimized to (0.33, 0.32)^24^. Li *et al*. fabricated the WLEDs by using the green and red emissive CsPbX_3_ QDs on a blue LED chip that leads to a tunable CCT in the range of 2500 K to 11,500 K and the color coordinates of (0.33. 0.30)^12^. The properties exhibited by the WLEDs based on halide perovskites are superior compared to the commercialized phosphor-based WLEDs, especially in regards to cost and color rendition^25^.

However, the lead commonly present in these materials currently precludes their practical utilization to a greater extent due to its toxicity. Therefore, the development of lead-free perovskites is the key to practical applications. As a result, much effort has been devoted to the development of lead-free or lead-reduced halide perovskites with suitable band gaps that are capable of preserving their optoelectronic properties by incorporating the non-toxic elements^26–29^. In this regard, the synthesis of lead-free perovskites using the tin (Sn^2+^) as a divalent cation with a potential application in solar cells has been demonstrated primarily, however, their poorer stability, smaller bandgap, and intrinsic defects yielding a low PLQY discourage further applications^30,31^. Jacobson *et al*. replaced Pb^2+^ completely by using Sr^2+^, however, the bandgap energy (E_g_ = 3.6 eV) was found to be too large to serve as photon down-conversion materials in WLEDs^32^. The lead-free double perovskites with A_2_BB′X_6_ structure (where ‘B’ is a monovalent cation and ‘B′’ is a trivalent cation) were also proposed as a candidate for the applications in optoelectronic devices^33–35^. These compounds have good stability, suitable bandgap energy, and are non-toxic in nature^33,36^. However, the double perovskites tend to have an indirect bandgap, which is not favorable for the applications in optoelectronic devices^36,37^.

The incorporation of the homovalent or heterovalent metal cations at the B-site of halide perovskites to partially replace the Pb^2+^ cations has also been explored as an alternative approach to reducing the toxicity of lead and make them environmental-friendly^38–41^. Recently, we reported the synthesis of lead-reduced CsPb_1-x_Mg_x_X_3_ (up to x = 20%) NCs for the fabrication of the WLEDs^42^. Our experimental results showed that the as-synthesized NCs retained the superior optical properties and these NCs have been utilized to fabricate WLEDs with superior color quality. Partial replacement of Pb^2+^ using Zn^2+^ in lead halide perovskites and their potential applications in photovoltaic cells have been demonstrated^43–45^. Besides the reduction of lead content, the as-fabricated solar cells resulted in enhanced thermal stability and improved photovoltaic performance of the device structure. The partial substitution of the reduced B-site cations (compared to Pb^2+^) such as the transition metal ions can reduce the size of eight cubo-octahedral voids which has the potential to decrease all of the cubo-octahedral voids for A-site cations in the ABX_3_ lattice. This results in a stable perovskite phase with superior thermal stability^38,45^. Furthermore, Shen *et al*. observed that the application of Zn^2+^-alloyed CsPbI_3_ NCs as a light emitter resulted in an LED with a peak external quantum efficiency of 15.1%^46^. However, there is no report on the fabrication of WLEDs using Zn^2+^-alloyed CsZn_x_Pb_1-x_X_3_ NCs as color conversion layers.

In this work, the comprehensive studies are carried out to investigate the effect of Zn^2+^-doping on the structural and optical properties of CsZn_x_Pb_1-x_X_3_ NCs (x up to 15%). The ultimate goal of this work is to realize the low-cost, highly efficient, and environmental-friendly white light with superior color quality. The inclusion of Zn^2+^ in the CsZn_x_Pb_1-x_X_3_ NCs results in a lattice contraction, however, the NCs maintain identical crystal structure and the morphology. The as-grown NCs exhibit strong photoluminescence (PL) emissions that are blue-shifted with increasing Zn^2+^ content because of the reduced size of unit cells while maintaining the high PLQY (>16% for CsZn_x_Pb_1-x_Cl_3_ and >80% for CsZn_x_Pb_1-x_Br_3_). In addition, the tunable bandgap from 1.82 eV to 3.19 eV and tunable emission from 411 nm to 636 nm are obtained by engineering the halide compositions for 15% Zn^2+^ inclusion. Furthermore, the prototype WLEDs based on CsZn_x_Pb_1-x_X_3_ NCs (for 15% Zn^2+^) are experimentally demonstrated by stacking the Zn^2+^-alloyed blue-, green-, yellow-, and red-emitting NCs on top of a UV LED. The WLEDs emit white light with a tunable CCT from warm to cold (2218–8335 K). In addition, an extra high CRI (maximized to 93) and high LER (268–318 lm·W^−1^) are achieved which is superior to the previous studies on WLEDs based on metal halide perovskites doped with other transition metals. Thus, this work opens up new prospects to engineer the properties of compositionally diverse lead-reduced halide perovskite NCs potentially to be used in lighting technology.

## Results and Discussion

### Effect of Zn^2+^-alloying on the Structural, Morphological, and Optical Properties of CsZn_x_Pb_1-x_Cl_3_ and CsZn_x_Pb_1-x_Br_3_ NCs

**Figure 1:**
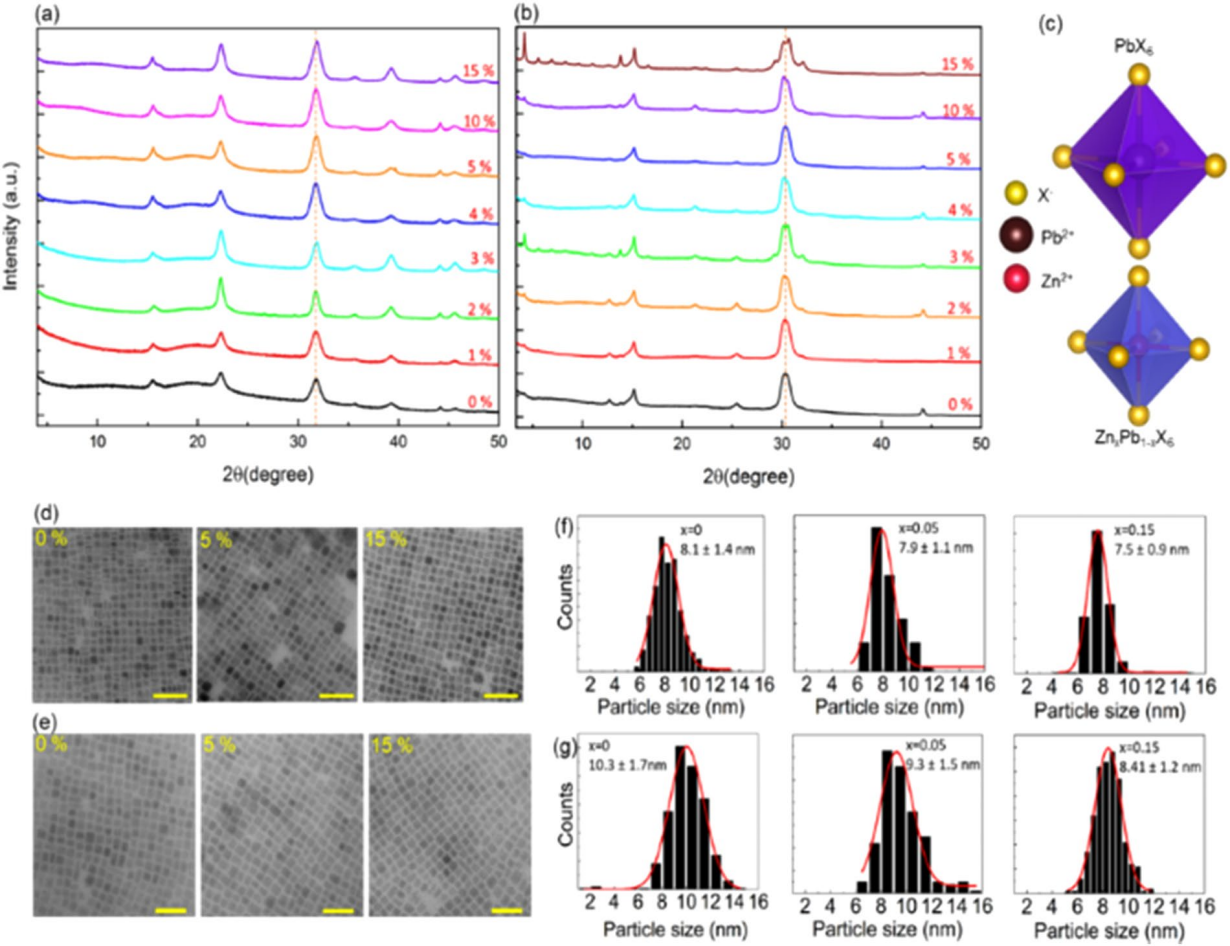
Structural and morphological properties of CsZn_x_Pb_1-x_X_3_ NCs at a different content of Zn^2+^. (**a,b**) XRD patterns of CsZn_x_Pb_1-x_Cl_3_ and CsZn_x_Pb_1-x_Br_3_ NCs. (**c**) Schematic overview of contraction of octahedral after partially replacing Pb^2+^ by smaller Zn^2+^ ions. (**d,e**) TEM images (scale bar, 50 nm) of the NCs. (**f,g**) Histograms showing size distribution of corresponding NCs.

The effect of the inclusion of Zn^2+^ on the structural property of CsPbCl_3_ and CsPbBr_3_ is investigated by measuring the XRD patterns. The XRD patterns of CsZn_x_Pb_1-x_Cl_3_ (Fig. 1a) and CsZn_x_Pb_1-x_Br_3_ (Fig. 1b) for the different concentrations of Zn^2+^ confirm that the NCs have cubic structure and the phase is independent of Zn^2+^ content. The main diffraction peaks are at ∼15°, ∼22°, ∼30°, and ~38° corresponding to the (100), (110), (200), and (211) diffraction planes of the cubic crystal structure, respectively. However, there is some evidence that the structure of the CsPbCl_3_ and CsPbBr_3_ NCs exhibit an orthorhombic phase at the unit cell level^47^. The opposed orthorhombic subdomains of the NCs annihilate their superstructural peaks leading to a perceived cubic phase that is in reality pseudocubic. As shown in Fig. 1b, the diffraction peak corresponding to the angle ∼30° for CsZn_x_Pb_1-x_Br_3_ appears more discrete with a gradual increase in the content of Zn^2+^. This is an indication of the presence of the orthorhombic structure due to the octahedral tilting^47,48^. Furthermore, the Zn^2+^-alloyed CsZn_x_Pb_1-x_Cl_3_ and CsZn_x_Pb_1-x_Br_3_ NCs exhibit the diffraction peaks shifted towards a higher 2θ range. The shift corresponds to 0.174° and 0.276° for CsZn_x_Pb_1-x_Cl_3_ and CsZn_x_Pb_1-x_Br_3_ NCs, respectively when the concentration of Zn^2+^ increases form 0% to 15% (Supplementary Fig. S1). The observed peak shift is due to the lattice contraction of as-synthesized NCs within the unit cell. The size of the lattice decreases from 5.632 Å to 5.601 Å for CsZn_x_Pb_1-x_Cl_3_ NCs and from 5.869 Å to 5.817 Å for CsZn_x_Pb_1-x_Br_3_ NCs. The lattice contraction suggests that Pb^2+^ cations with a bigger ionic radius (119 pm) occupying the B-site of BX_6_ octahedra are being partially replaced by Zn^2+^ cations with a smaller ionic radius (74 pm) at the same lattice site of Pb^2+^. If Zn^2+^ atoms have resided in interstitial sites, we would have observed a lattice expansion with increasing Zn^2+^ content^46^. Thus, the incorporation of the smaller guest element Zn^2+^ in the octahedra of parent CsPbX_3_ perovskite structure results in the reduced size of the BX_6_ octahedra (Fig. 1c) that gives rise to the distortion and tilting of the BX_6_ octahedra. The octahedral tilting accompanied by the change in the lattice constant lead to the increased bandgap and blue-shifted PL emission at higher content of Zn^2+^ which will be discussed in the latter part. Interestingly, the XRD scans do not indicate the presence of the additional peaks even after the incorporation of guest cation Zn^2+^, signifying the absence of any crystalline impurities. Furthermore, the presence of a low angle (<10°) peak in the XRD patterns indicate the presence of superlattice structures. The intensity of the superstructural peaks increases with an increase in the content of Zn^2+^ which indicates the enhancement in the periodicity of the NCs.

The morphology of the as-synthesized NCs is investigated using transmission electron microscopy (TEM). The TEM images (Fig. 1d,e) show the uniformly distributed nanocubes without a change in morphology even after the inclusion of Zn^2+^ in the parent NCs. However, the average side length of CsZn_x_Pb_1-x_Cl_3_ NCs decreased from ∼8.1 ± 1.4 nm to ∼7.5 ± 0.9 nm and that of CsZn_x_Pb_1-x_Br_3_ decreased from ∼10.3 ± 1.7 nm to ∼8.41 ± 1.2 nm when the content of Zn^2+^ is increased from 0% to 15%. The decrease in particle size at high content of Zn^2+^ may be a result of increased halide ion uptake, which hinders the crystal growth^42,46^. The size distribution analysis of as-grown samples is illustrated in Fig. 1f,g. From these histograms, it can be concluded that the particle size distribution also decreases with increasing the content of Zn^2+^. Thus, from the XRD and TEM analysis, we found that the incorporation of Zn^2+^ results in the increase in periodicity and uniformity of the NCs with a decrease in NCs size.

**Figure 2:**
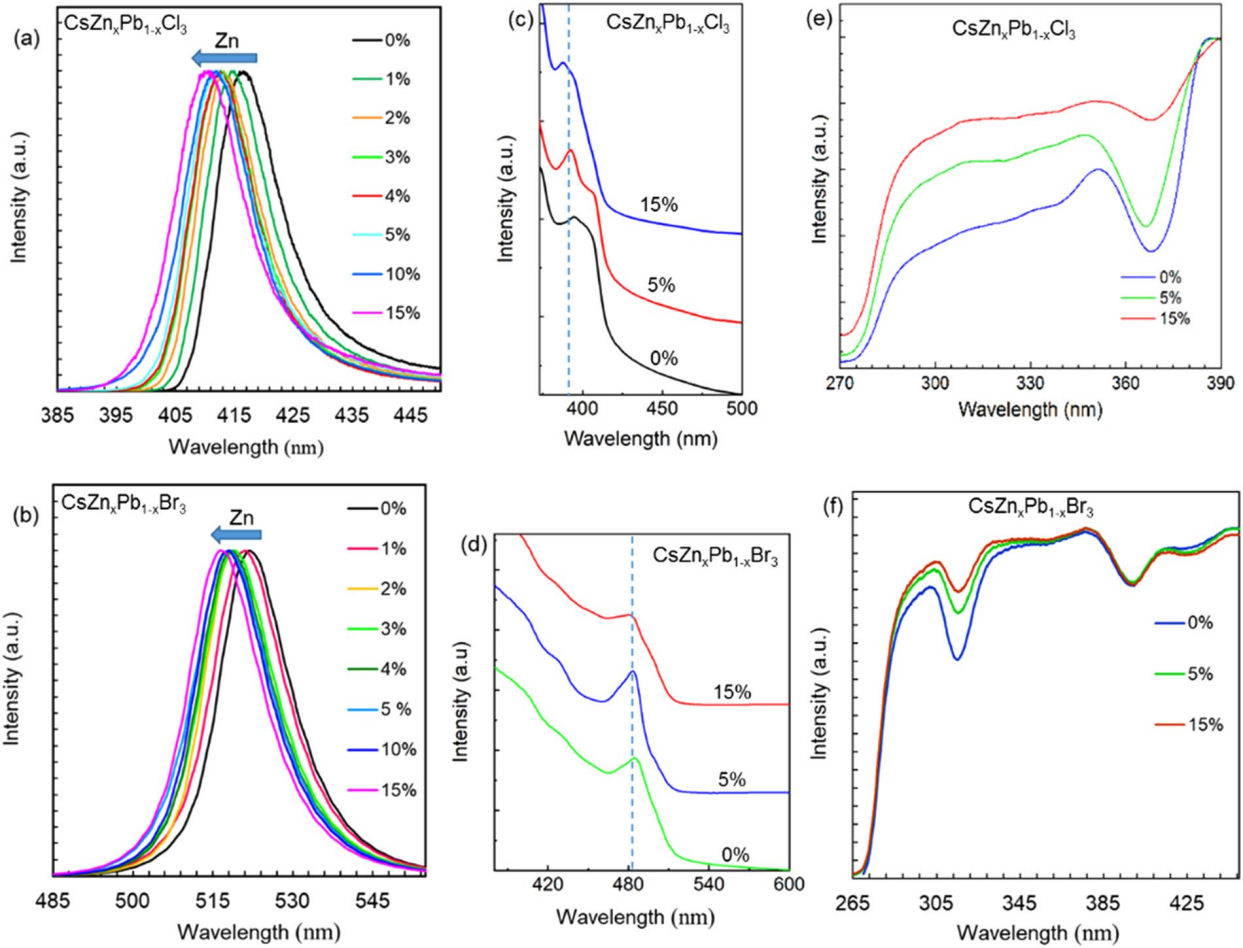
Optical properties of CsZn_x_Pb_1-x_X_3_ NCs at a different content of Zn^2+^. (**a,b**) PL emission spectra of CsZn_x_Pb_1-x_Cl_3_ and CsZn_x_Pb_1-x_Br_3_ NCs, respectively. (**c,d**) UV-visible absorption spectra of the corresponding NCs. (**e,f**) Excitation spectra of the corresponding NCs.

**Figure 3:**
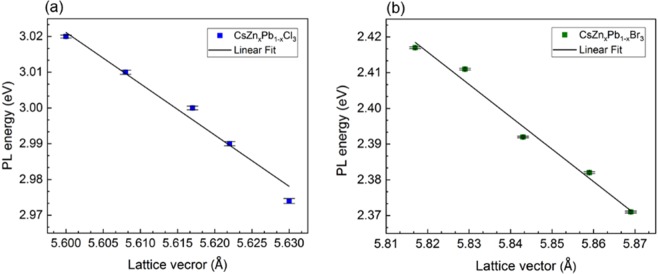
The variation of PL energy with lattice vector for different compositions of Zn^2+^, fitted with an error bar which is approximated using the Gaussian model fitting. (**a**) PL energy variation of CsZn_x_Pb_1-x_Cl_3_ NCs. (**b**) PL energy variation of CsZn_x_Pb_1-x_Br_3_ NCs. The different points correspond to 0%, 3%, 5%, 10%, and 15% of Zn^2+^, respectively. The highest energy point corresponds to the highest content of Zn^2+^ and the lowest energy point corresponds to the lowest content of Zn^2+^.

The effect of the inclusion of Zn^2+^ on the optical properties of CsPbX_3_ perovskites is characterized by measuring PL and absorption spectra as shown in Fig. 2. The normalized PL and absorption spectra of CsZn_x_Pb_1-x_Cl_3_ and CsZn_x_Pb_1-x_Br_3_ exhibit a modest blue-shift with increasing Zn^2+^ content in the host materials (Fig. 2a–d). Since both the Pb^2+^ and Zn^2+^ precursors contain the same halides, this paves a pathway for the inclusion of Zn^2+^ ions upon its introduction into the host CsPbX_3_ matrix and favors the change in the optical properties of Zn^2+^-alloyed NCs. As-grown CsZn_x_Pb_1−x_Cl_3_ NCs exhibit PL emission in the violet region with emission peak attenuated from 417 nm to 411 nm (excited at 300 nm), whereas CsZn_x_Pb_1-x_Br_3_ NCs, show the green emission which is tuned from 522 nm to 517 nm (excited at 365 nm). This blue-shift in the optical properties is related to the slight decrease in the size of the NCs that can be ascribed to the weak quantum confinement effect. The full width at half-maximum (FWHM) of PL emissions of CsZn_x_Pb_1-x_Cl_3_ NCs is decreased from 13.4 nm to 12.8 nm and that of CsZn_x_Pb_1-x_Br_3_ NCs is decreased from 16.9 nm to 15.5 nm when the content of Zn^2+^ is increased from 0% to 15%. The narrow FWHM shows that the obtained NCs are uniformly distributed and decreased FWHM indicates an increase in the uniformity in the size distribution of NCs. This observation is also in agreement with the TEM images. In addition, the reduced FWHM of PL emission is advantageous for the fabrication of energy-efficient LEDs with high color purity. The UV-visible absorption spectra show a well-defined absorption peak for all the samples

(independent of the halide) that indicate the larger excitonic binding energy of the NCs^49^. The key spectral features of the parent CsPbCl_3_ and CsPbBr_3_ NCs, such as the sharp optical absorption edge and well-defined absorption peaks are maintained even after the inclusion of Zn^2+^ (Fig. 2c,d). The absorption peak of CsZn_x_Pb_1-x_Cl_3_ observed at ∼394 nm is blue-shifted to ∼388 nm and that of CsZn_x_Pb_1-x_Br_3_ is shifted from ∼485 nm to ∼480 nm. The corresponding absorption peak was examined to estimate the optical bandgap of the different composite of NCs. The bandgap of CsZn_x_Pb_1-x_Cl_3_ and CsZn_x_Pb_1-_xBr_3_ NCs is tuned from 3.144 eV to 3.194 eV and 2.557 eV to 2.578 eV, respectively. The increase in bandgap is most likely due to the increasing electronegativity (χ) of the Zn^2+^ ions (χ ∼1.7) occupying the B-site in the ABX_3_ perovskite structure compared to the Pb^2+^ ions (χ ∼1.6), which reduces the dimensions for both the crystal lattice and the NC and is in agreement with the XRD and TEM analyses^50^. Furthermore, all the samples prepared with various Zn^2+^ content exhibit identical and broadband excitation spectra (Fig. 2e,f). The excitation spectra are measured by monitoring the corresponding peak emission wavelength. The broad spectral band suggests that these perovskite materials can be effectively excited at a broad range of wavelengths. The excitation spectrum is at a higher energy region compared to the corresponding emission energy spectrum that reflects the potential applications of these materials in conjunction with III-nitride based UV/blue LEDs to obtain white light through photon down-conversion. The dual spectral bands shown in the excitation spectra are related to a charge-separated bandgap state (at higher wavelength) and the charge transfer band (at lower wavelength)^51^. Furthermore, the observed linear relationship between the PL energy and the lattice vector (Fig. 3a,b) for the NCs with the different incorporated amounts of Zn^2+^ signifies that the effect of lattice contraction is dominant factors in altering the observed optical behavior of the as-synthesized NCs. In brief, the lattice contraction favors the stronger interactions between ‘B’ and ‘X’, which induces the antibonding combinations between Pb(6p) and Br(4p) resulting in a shift of the conduction band minimum to higher energies and, hence the bandgap of NCs increases^39^. A similar trend was observed on ABX_3_ NCs (with tunable blue-shifted PL emission) that were obtained by other compositional control^3,20^. Moreover, the as-grown NCs exhibit the high PLQYs as shown in Table 1. The PLQY of CsZn_x_Pb_1-x_Cl_3_ NCs at 0% Zn^2+^ is 19% and that at 15% Zn^2+^ is 16%. Similarly, PLQY of CsZn_x_Pb_1-x_Br_3_ NCs at 0% Zn^2+^ is 93% and that at 15% Zn^2+^ is 92%. The PLQY of CsZn_x_Pb_1-x_Cl_3_ NCs is reduced by a higher value in comparison to CsZn_x_Pb_1-x_Br_3_ NCs. The halide vacancies and Cs vacancies are the dominant defect states for CsPbCl_3_ and CsPbBr_3_ perovskite NCs. At the highest content of Zn^2+^ doping, the formation energy corresponding to these vacancies in the Cl-based NCs might have decreased by a larger amount in comparison to the Br-based NCs. Eventually, this results in an increase in the structural defects leading to a higher decrease in PLQY of the Cl-based NCs^52,53^. This demonstrates that Zn^2+^ has an impact on absolute PLQYs of alloyed NCs suggesting that the inclusion of Zn^2+^ altered the defect states of the host NCs.

**Table 1:** The PLQYs of CsZn_x_Pb_1-x_Cl_3_ and CsZnxPb_1-x_Br_3_ at various Zn^2+^ content.

Zn^2+^ content x (%)	PLQY (%)	
	CsZn_x_Pb_1-x_Cl_3_	CsZn_x_Pb_1-x_Br_3_
0	19	93
2	16	81
5	16	91
10	15	80
15	16	92

### Exploration of Zn^2+^-alloyed CsZn_x_Pb_1-x_I_3_ Perovskites

The study of the effect of Zn^2+^ incorporation on the iodide-based perovskite NCs is carried out. XRD patterns (Supplementary Fig. S2) indicate that the samples prepared and stored in an ambient atmosphere decomposed significantly after some hours, similar to the study reported earlier^54^. This is ascribed to the degradation of the NCs from the black phase to the non-emissive yellow phase. However, the fresh samples prepared without and with the inclusion of Zn^2+^ show tunable PL emission (672–646 nm) with the FWHM in the range of 50–54 nm, Supplementary Fig. S3.

### Tunable Emission from CsZn_0.15_Pb_0.85_X_3_ (X = Cl^−^, Br^−^, I^−^) NCs by Varying the Halide Compositions

**Figure 4:**
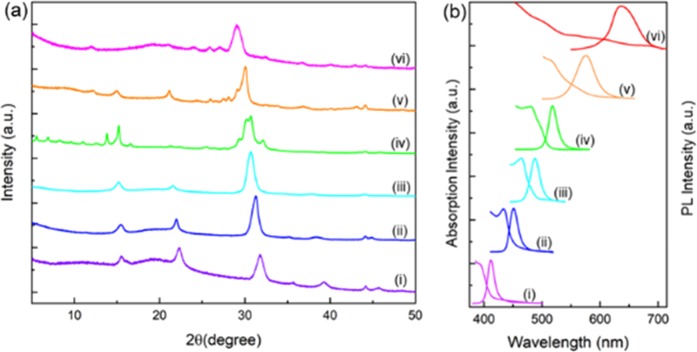
Structural and optical properties of mixed halides NCs at 15% Zn^2+^. (**a**) XRD patterns of CsZn_0.15_Pb_0.85_X_3_ NCs according to ‘X’ as- (i) Cl^−^, (ii) Cl^−^:Br^−^ = 1:1, (iii) Cl^−^:Br^−^ = 1:3, (iv) Br^−^, (v) Br^−^:I^−^ = 1:1, and (vi) Br^−^:I^−^ = 1:3. (**b**) The PL emission and the UV-visible absorption spectra corresponding to the as-synthesized NCs.

All-inorganic halide perovskite NCs (15% Pb^2+^-reduced) with tunable emission from blue to red is synthesized through the compositional modulation of halide ions that results in a series of CsZn_0.15_Pb_0.85_(Cl_x_Br_1-x_)_3_ and CsZn_0.15_Pb_0.85_(Br_y_I_1-y_)_3_ NCs. As revealed by the XRD patterns (Fig. 4a) and the TEM images (Supplementary Fig. S4) these mixed-halide NCs confirm that they possess identical crystalline structure and morphology except for the slight changes in the dimension of the NCs. The PL emission and the corresponding UV-visible absorption measurements indicate that the mixed halides NCs experience a red-shift in their peak position on decreasing the ratio of Cl^−^/Br^−^ and Br^−^/I^−^ in CsZn_0.15_Pb_0.85_(Cl_x_Br_1-x_)_3_ and CsZn_0.15_Pb_0.85_(Br_y_I_1-y_)_3_ NCs respectively as shown in Fig. 4b. The PL emission is engineered across the significant portion of the visible spectrum (411–636 nm) with narrow FWHM (13–43 nm) and PLQY above 16%, which is summarized in Supplementary Table S1. The corresponding bandgap of the as-prepared NCs can be finely tuned from 3.19 eV to 1.82 eV. A linear absorption profile for the higher I^−^ content in the CsZn_0.15_Pb_0.85_(Br_y_I_1-y_)_3_ NCs indicates the formation of larger NCs which is in agreement with the TEM images provided in Supplementary Fig. S4.

Furthermore, the remarkable fluorescence quality of halide perovskite NCs such as narrower line-width and wider color gamut coincide with the requirements for high-quality white light generation. However, metal halide perovskite NCs having different emissions cannot directly be mixed to produce white light as cadmium-based NCs, because of the rapid anion exchange process for this material. To overcome this issue, Pathak *et al*. have reported the remote phosphor technique to obtain white light by stacking different color emitting NCs containing quartz substrates as the color conversion layers on top of the blue LEDs and obtained a CRI of 86 and a CCT of 5229 K^55^. In this structure, the gap maintained between the LED chips and the conversion layers protect the NCs from the direct effect of heat produced by the LEDs. This non-contact configuration is a reliable approach to avoid the heating effect on the NCs without disturbing the LED junction principle. Thus, this work stimulates us to study further about the applications of this technique. We proposed the tetradic phosphor technique to get white light by employing the four different color emitting NCs in conjunction with UV LED and succeed to observe a high value of CRI (up to 95.4) and LER (>300 lm·W^−1^) with tunable CCT from warm to cold hue^56,57^. The enhancement in the quality and optical property of light is attributed to the incorporation of an additional yellow phosphor component that bridges the energy gap between the green and red phosphor components.

**Figure 5:**
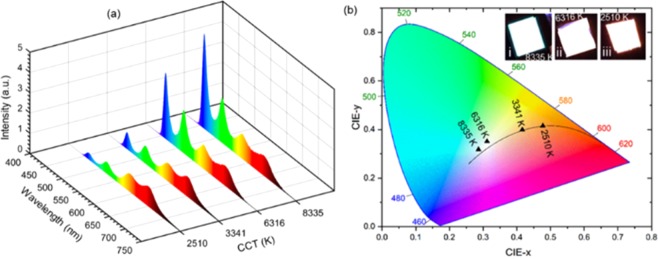
Optical characterization of white light emission. (**a**) EL spectra of white light with a tunable CCT. (**b**) CIE diagram showing CIE coordinates of white light with different CCT. (Inset is the photographs of the devices at CCT: (i) 8335 K, (ii) 6316 K, and (iii) 2510 K of white light.)

Herein, we have used the lead-reduced CsZn_0.15_Pb_0.85_X_3_ NCs to fabricate the fourfold phosphor-based WLEDs employing a remote phosphor technique. For this configuration, we have prepared the thin films of individual color such as blue [λ_peak_ ∼451 nm, CsZn_0.15_Pb_0.85_(Cl_0.5_Br_0.5_)_3_], green [λ_peak_ ∼517 nm, CsZn_0.15_Pb_0.85_Br_3_], yellow [λ_peak_ ∼576 nm, CsZn_0.15_Pb_0.85_(Br_0.5_I_0.5_)_3_], and red [λ_peak_ ∼636 nm, CsZn_0.15_Pb_0.85_(Br_0.25_I_0.75_)_3_] that acts as color conversion layers. Then, we stacked them on top of the commercialized UV LED chip to obtain white light emission by injecting the current into UV LED chips (λ_peak_ ∼405 nm, runs at 8 V and 10 mA). The tunable CCT was obtained by tuning the ratios of blue and green to yellow and red emissions to achieve the different spectral power distributions of white light as reported in our previous work^56,58^. Here we have engineered the amount of blue-emitting, green-emitting, yellow-emitting nanocrystals used in the fabrication of color-emitting thin films to achieve the different spectral power distributions with the goal of obtaining the white light with tunable CCT. For instance, for obtaining the warm white, we increased the yellow- and red-emitting NCs in comparison to the blue- and green-emitting NCs whereas for the cool white light, we increased the amount of blue- and green-emitting NCs in comparison to the other two NCs. The EL spectra of as-fabricated WLEDs are measured which are shown in Fig. 5a. No intermediate EL emission peak is detected, suggesting that the anion exchange reactions have been eliminated. Then, we have calculated the CCT, CRI, LER, and CIE coordinates based on the EL spectrum of individual WLEDs (shown in Table 2). The tunable CCT from warm to cold white (2218–8335 K) is achieved with high CRI (84–93) and LER (268–318 lm·W^−1^). The maximum CRI we obtained is 93 at a CCT of 2218 K and an LER of 286 lm·W^−1^. These CRI and LER are superior to the previous reports on WLEDs based on CsPbX_3_ NCs doped with other transition metal ions^41,59^. The CIE diagram is shown in Fig. 5b. The inset of Fig. 5b is the photographs of the as-fabricated WLEDs having different CCT at 8335 K, 6316 K, and 2510 K. The EL spectra (Fig. 5a) demonstrates that a relatively high- the integrated intensity of the yellow and red phosphors is suitable for warm white light whereas increasing the integrated intensity of blue and green phosphors cool the light hue. The warm white creates a relaxing and cozy atmosphere whereas the neutral and cold white produces energizing and uplifting effects on human health. Besides, the comparable high CRI and LER are due to the smaller FWHM of the emission. The high CRI of light source indicates that the human eyes can recognize the natural color of the object under the light source and the high value of LER demonstrates the high vision performance which will lead to fabricating the highly energy-efficient LEDs. The CIE coordinates of the corresponding white light are close to the Planckian locus (Fig. 5b), which suggests that the color temperature we obtained is pleasant for human eyes. In addition, one of the WLED (CCT of 5572 K) shows the CIE coordinates at (0.33, 0.36) is close to the CIE coordinates of standard neutral white light (0.33, 0.33). Thus, these remarkable optical properties of WLEDs based on Zn^2+^-alloyed all-inorganic lead halide perovskites make them promising for light-emitting devices as a replacement for conventional phosphors.

**Table 2:** Estimation of optical parameters of WLEDs.

CCT (K)	CRI	LER (lmW^−1^)	CIE-x	CIE-y
2218	93	286	0.5088	0.4270
2510	89	281	0.4772	0.4154
3341	90	283	0.4162	0.3992
4801	89	318	0.3580	0.4128
5572	90	300	0.3307	0.3648
6316	88	287	0.3115	0.3514
8335	84	268	0.2862	0.3180

The table represents the measured value of CCT, CRI, LER, and CIE coordinates of as-fabricated WLEDs.

## Conclusion

In summary, a series of high-quality Zn^2+^-alloyed CsZn_x_Pb_1-x_X_3_ NCs are synthesized using the ZnX_2_ salts. Since both the Pb^2+^ and Zn^2+^ precursors contain the same halide, this paves a pathway for the inclusion of Zn^2+^ upon its introduction in the host CsPbX_3_ matrix. When the content of Zn^2+^ is increased from 0% to 15% in CsZn_x_Pb_1-x_X_3,_ the NCs retain the identical crystal structure and morphology except for the lattice contraction and the reduced NCs dimension. The lattice contraction and the reduced unit cell dimension of the NCs resulted in an increased bandgap and blue-shifted PL emission without much reduction in the PLQYs (>16% for CsZn_x_Pb_1-x_Cl_3_ and >80% for CsZn_x_Pb_1-x_Br_3_). Moreover, owing to their outstanding optical properties, the CsZn_x_Pb_1-x_X_3_ NCs exhibit potential for use in WLEDs as a color conversion layer. Experimentally, for the first time, the fourfold conversion layers (blue, green, yellow, and red) of CsZn_0.15_Pb_0.85_X_3_ NCs are used with UV LED chips to obtain white light having superior quality that leads to a tunable color hue (2218–8335 K) with CRI maximized to 93, and high LER in the range of 268–318 lm·W^−1^. Based on these results, we expect that these NCs are feasible for obtaining next-generation white light sources with remarkable optical properties and reduced environmental toxicity.

## Materials and Methods

### Synthesis of Zn^2+^-alloyed CsZn_x_Pb_1-x_X_3_ NCs

We used the hot-injection method with a slight modification for the synthesis of CsZn_x_Pb_1-x_X_3_ NCs^20,42^. Shortly, the preparation of *the* Cs-oleate precursor was performed by adding cesium carbonate (Cs_2_CO_3_, 0.243 g) to oleic acid (OA, 0.75 mL), and octadecene (ODE,12 mL) into 50 mL flask. The mixture was dried under vacuum for approximately 1 h at 120 °C and then heated under N_2_ to 150 °C until all Cs_2_CO_3_ had reacted with OA. Then, 0.188 mmol of PbX_2_ [PbBr_2_ (0.069 g), PbCl_2_ (0.052 g), and PbI_2_ (0.087 g)] or their mixtures (in desired ratio) and 0.188 mmol of ZnX_2_ [ZnBr_2_ (0.042 g), ZnCl_2_ (0.026 g), and ZnI_2_ (0.060 g)] were taken into 25 mL flask with ODE (5 mL), OA (0.5 mL), and oleylamine (0.5 mL) separately, and were dried using vacuum oven for 1 h at 120 °C. Note that the Trioctylphosphine (1 mL per 5 mL of ODE) was added into the aforementioned mixture for the synthesis of chloride-based NCs. These mixtures were stirred at 90 °C on a hot plate until the PbX_2_ and ZnX_2_ salts were completely dissolved. To the solution obtained by mixing the proportionate amount of PbX_2_ and ZnX_2_ precursors, the Cs-oleate solution (0.4 mL, preheated to 100 °C before injection) was quickly injected into the mixture. This was followed by the immediate transference of the mixture into the ice-water bath where the mixture was allowed to cool for a certain time (∼2 min). The cooling process results in the crude solution of the NCs that were subjected to centrifugation at 3500 rpm for 10 minutes. Then, the precipitated NCs were separated from the supernatant, thereby transferring the NCs in toluene for storage. In addition, the composition of halides was attenuated on CsZn_x_Pb_1-x_X_3_ (15% Zn^2+^) to obtain the desired tunable emission. Finally, the colloidal solution of the samples was used for the characterization and device fabrication.

### WLED fabrication

Commercially available GaN UV LED chips with the emission peak centered at 405 nm were purchased. The quartz substrates were ultrasonically cleaned in detergent, isopropyl alcohol, deionized water, and ethanol for 15 minutes in sequence and dried naturally. The CsZn_x_Pb_1-x_X_3_ perovskite NCs active layers were spin-casted from their colloidal solution at 1000 rpm for 60 seconds for different cycles (10 µL/cycle) which is then dried at an ambient atmosphere without providing any external heat. The four different emissive layers (blue, green, yellow, and red) prepared in separate quartz substrates were stacked one above another in the hierarchy of their increasing energy order in conjunction with UV LED chip to obtain white light.

### Characterization

The characterization techniques that we had performed were similar to our previous report^42^. In details, to study the structural properties of the samples, the XRD measurements were performed using A Rigaku SmartLab diffractometer with CuKα_1_ radiation, λ = 1.54 Å operated at 40 kV and 44 mA, and at an angular range (2θ) with a step of 0.01°. The TEM images recorded by a Hitachi H-7000 transmission microscope operated at 75 kV were used to study the morphology of the samples. The PL spectra were recorded using a spectro-fluorophotometer (Shimadzu, RF6000). UV-visible absorption spectra were obtained using a Varian Carry 50 Scan UV spectrophotometer. The PLQYs were measured using a QE-pro spectrometer (QEP02037, Ocean Optics) and an integrated sphere (819C-SF-6, Newport). The NCs were excited using a laser source at a wavelength of 350 nm for chloride-based NCs and 405 nm for bromide and iodide-based NCs for PLQY measurement. The measurement of EL spectra was carried out using a QE-pro spectrometer (QEP02037, Ocean Optics) in conjunction with 405 nm UV LED chip and Keithley 2450 source units operated at 8 V and 10 mA under forward bias.

## Supplementary information

Supplementary information

## Data Availability

All data needed is provided in the paper and the Supplementary Materials to evaluate the conclusions of our work. Additional data related to this paper may be requested from the authors.
