# Supplementary material for: Zn-Alloyed All-Inorganic Halide Perovskite-Based White Light-Emitting Diodes with Superior Color Quality
Source: Sci Rep. 2019 Dec 9;9:18636. doi: 10.1038/s41598-019-55228-1 (PMC6901607; doi:10.1038/s41598-019-55228-1)
Supplement: Supplementary file 1 — Supplementary information [file 41598_2019_55228_MOESM1_ESM.pdf]

Supplementary Information

Zn-Alloyed All-Inorganic Halide Perovskite-Based White Light-Emitting Diodes with Superior Color Quality

Saroj Thapa, Gopi Chandra Adhikari, Hongyang Zhu, Alexei Grigoriev, and Peifen Zhu

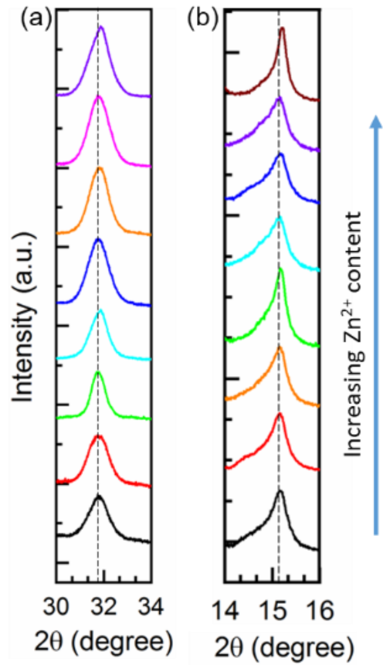

Figure S1. The magnified XRD patterns of the $CsZn_xPb_{1-x}X_3$ NCs indicating the shift in the peak position at a particular diffraction angle. (**a**)The diffraction peak shift corresponding to $CsZn_xPb_{1-x}Cl_3$ NCs. (**b**) The diffraction peak shift corresponding to $CsZn_xPb_{1-x}Br_3$ NCs.

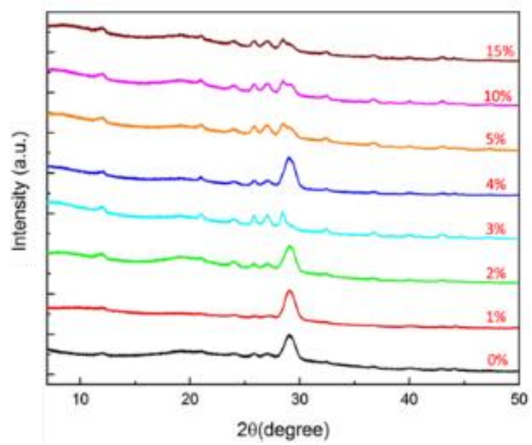

Figure S2. Structural characterization of iodide-based NCs. The spectra represents the XRD patterns of $CsZn_xPb_{1-x}I_3$ NCs synthesized at different content of Zn^{2+}.

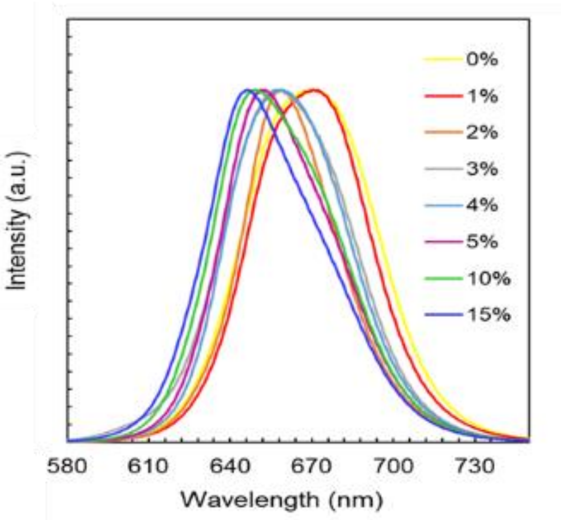

Figure S3. Optical characterization of iodide-based NCs. The spectra indicates the tunable PL emission peak of $CsZn_xPb_{1-x}I_3$ NCs at different content of Zn^{2+}.

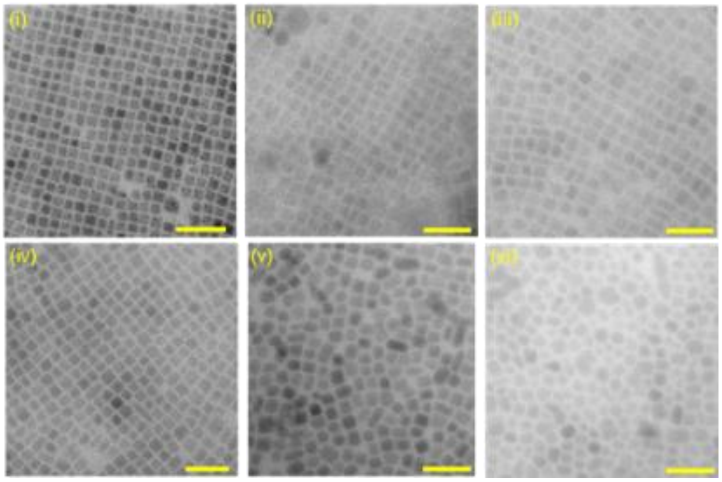

Figure S4. Morphological characterization of mixed halides NCs at 15 % of Zn^{2+}. The images refers to the TEM images of $CsZn_{0.15}Pb_{0.85}(X)_3$ NCs for the different compositions of halides according to 'X' as- (i) Cl^-, (ii) Cl^-:Br^-=1:1, (iii) Cl^-:Br^-=1:3, (iv) Br^-, (v) Br^-:I^-=1:1, and (vi) Br^-:I^-=1:3. Scale bar is 50 nm.

Table S1. Estimation of optical parameters of mixed halides NCs. The table represents the measured value of PL peak, FWHM, bandgap, and PLQY of as-grown NCs.

Compound	PL peak (nm)	FWHM (nm)	Bandgap (eV)	PLQY (%)
$CsZn_{0.15}Pb_{0.85}(Cl)_3$	411	12.9	3.19	16
$CsZn_{0.15}Pb_{0.85}(Cl_{0.5}Br_{0.5})_3$	451	19.5	2.85	42
$CsZn_{0.15}Pb_{0.85}(Cl_{0.25}Br_{0.75})_3$	488	20.3	2.66	59
$CsZn_{0.15}Pb_{0.85}(Br)_3$	517	15.5	2.57	92
$CsZn_{0.15}Pb_{0.85}(Br_{0.5}I_{0.5})_3$	576	34.7	2.39	71
$CsZn_{0.15}Pb_{0.85}(Br_{0.25}I_{0.75})_3$	636	43.3	1.82	78
